# Fine dissection of the tarsal tunnel in 60 cases

**DOI:** 10.1038/srep46351

**Published:** 2017-04-11

**Authors:** Y. Yang, M. L. Du, Y. S. Fu, W. Liu, Q. Xu, X. Chen, Y. J. Hao, Z. Liu, M. J. Gao

**Affiliations:** 1 Medical student in clinical medicine, Dalian Medical University, Dalian 116044, People’s Republic of China; 2 Department of Anatomy, Dalian Medical University, Dalian 116044, People’s Republic of China

## Abstract

The fine dissection of nerves and blood vessels in the tarsal tunnel is necessary for clinical operations to provide anatomical information. A total of 60 feet from 30 cadavers were dissected. Two imaginary reference lines that passed through the tip of the medial malleolus were applied. A detailed description of the branch pattern and the corresponding position of the posterior tibial nerve, posterior tibial artery, medial calcaneal nerve and medial calcaneal artery was provided, and the measured data were analyzed. Our results can be summarized as follows. **I**. A total of 81.67% of the bifurcation points of the posterior tibial nerve, which was divided into the medial and lateral plantar nerves, were located within the tarsal tunnel, not distal to the tarsal tunnel. **II**. The bifurcation points of the posterior tibial artery were all located in the tarsal tunnel. Almost all of the bifurcation points of the posterior tibial artery were lower than those of the posterior tibial nerve. The bifurcation point of the posterior tibial artery situated distal to the tarsal tunnel was not found. **III**. The number and the origin of the medial calcaneal nerves and arteries were highly variable.

## Introduction

The tarsal tunnel is a fibro-osseous structure that has a hard and poorly scalable wall. It belongs to the soft tissue space that has been used as route access in microinvasive and microsurgical operations^1^,^2^. The flexor retinaculum forms the medial wall of the tarsal tunnel and constitutes the lower and upper boundary of the tunnel. The medial wall of the talus and calcaneus and the distal medial wall of the tibia constitute the bottom. The medial malleolus is in front of the tunnel. The contents within the tarsal tunnel from the anterior medial to the posterior lateral side include the following: the posterior tibial tendon, the flexor digitorum longus tendon, the posterior tibial artery and veins, the posterior tibial nerve and the flexor hallucis tendon^3^,^4^,^5^,^6^,^7^,^8^,^9^. The posterior tibial nerve courses posterior and inferior to the medial malleolus and further divides into two main branches, the medial and lateral plantar nerves. The position of the bifurcation is variable. The bifurcation of the posterior tibial nerve determines its running direction, which is closely related to its clinical function. Some researchers have found that most of the bifurcation points are located in the tarsal tunnel, and few are located proximal or distal to the tarsal tunnel^10^,^11^,^12^. However, further knowledge is still needed, especially in Chinese patients.

The medial calcaneal nerve originates from the posterior tibial nerve and its branches and is considered to innervate the skin of the posterior medial arch of the foot. Its distribution has very important significance in the diagnosis and treatment of heel pain, tarsal tunnel syndrome, soft tissue and bone joint injury and heel pain secondary to neuroma heel pain^13^. The origin of the medial calcaneal nerve is highly variable; it can emerge from the posterior tibial nerve and/or the lateral plantar nerve. Some studies mentioned that this nerve could also originate from the medial plantar nerve, cross blood vessels in the tarsal tunnel and innervate the heel^12^,^14^. The number of medial calcaneal nerves is changeable, normally ranging from one to four^13^,^14^,^15^,^16^ but as many as five have been reported^17^.

The branch pattern of blood vessels in the tarsal tunnel is similar to the nerve, and the posterior tibial artery was also divided into two main branches, the medial and the lateral plantar arteries. The bifurcation point of the posterior tibial artery is distal to that of the posterior tibial nerve^11^. The posterior tibial artery and/or its branches form the medial calcaneal artery. The relevant reports on this artery and its branch pattern are relatively few in number.

In our research, the position of the bifurcation points and the branch pattern of the posterior tibial nerve and posterior tibial artery were studied. The position of the origination points and branch pattern of the medial calcaneal nerve and medial calcaneal artery were also recorded. At the same time, we also observed the location relationship between the posterior tibial nerve and the posterior tibial artery. Combined with previously reported methods, our research provides a fine anatomical basis for clinical operations and nerve block related to the tarsal tunnel.

**Figure 1:**
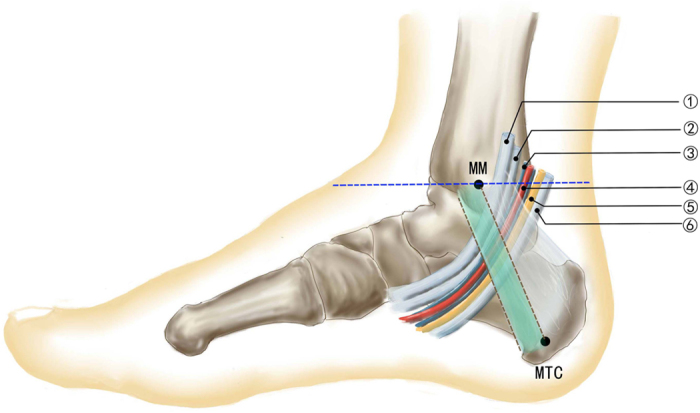
The structures within the tarsal tunnel with the reference lines applied. MM: medial malleolus. MTC: medial tubercle of the calcaneal. The blue horizontal line is Line A, which crosses the tip of the medial malleolus. The green oblique line (band) is Line B, with a 1 cm width, which spans from the tip of the medial malleolus to the medial tubercle of the calcaneus. This axis also represents the inferior edge of the flexor retinaculum and consequently the tarsal tunnel. ① Posterior tibial tendon, ② flexor digitorum longus tendon, ③ posterior tibial artery, ④ vein accompanying the artery, ⑤ posterior tibial nerve, ⑥ flexor hallucis tendon.

Based on past studies, with clearly defined anatomic structures of the tarsal tunnel as mark, two reference lines were set as shown in Fig. 1. Line A was a horizontal line that passed the tip of the medial malleolus^18^. Line B, with a width of 1 cm, was a line from the tip of the medial malleolus to the medial tubercle of the calcaneus^11^. A detailed description of the two reference lines can be found in following materials and methods section.

### Posterior tibial nerve

**Figure 2:**
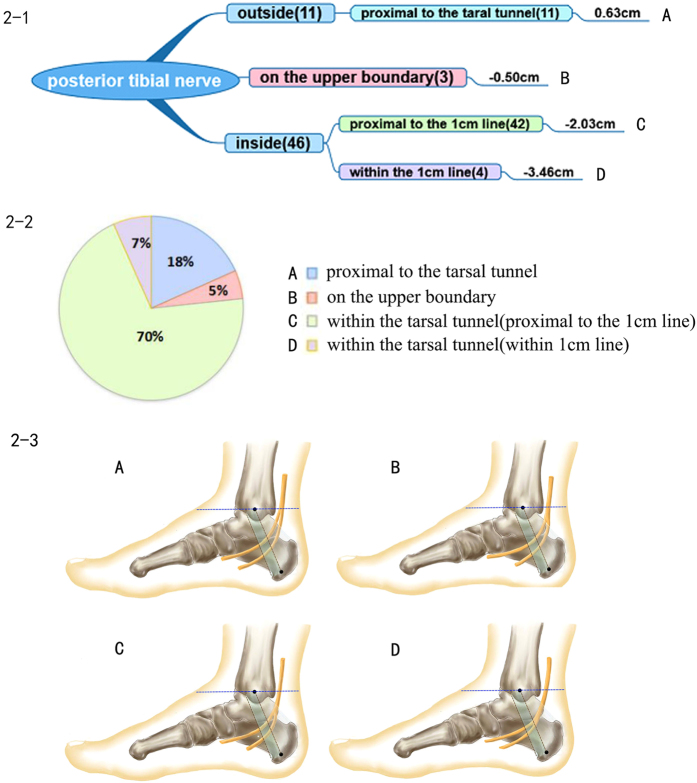
Posterior tibial nerve. 2–1 Representation of the branch pattern of the posterior tibial nerve. 2–2 Pie chart of the proportion of posterior tibial nerve branch patterns. 2–3 Diagram of each branch pattern. (**A**) Type I, bifurcation point of the posterior tibial nerve proximal to the tarsal tunnel. (**B**) Type II: bifurcation point just situated on the upper boundary of the tarsal tunnel. (**C**) Type III: bifurcation point located within the tarsal tunnel and proximal to the 1 cm reference Line B. (**D**) Type IV: bifurcation point within the tarsal tunnel also located in the area of the 1 cm reference Line B.

The posterior tibial nerve was divided into two major branches, the medial and lateral plantar nerves. The medial calcaneal nerve originated from the posterior tibial nerve and/or its branches. There were four types of branch patterns based on the bifurcation point position. Type I: the bifurcation point of the posterior tibial nerve proximal to the tarsal tunnel was found in 11 (18.33%) of 60 cases. Type II: the bifurcation was situated on the upper boundary of the tarsal tunnel in a total of 3 cases (5%). Type III: this pattern is the most common one. Bifurcation was located within the tarsal tunnel and was proximal to the 1 cm reference Line B. There were 42 cases of this pattern; the proportion was 70%. Type IV: the bifurcation point was located in the area of the 1 cm reference Line B, which is the distal end of the tarsal tunnel. There were 4 cases of this pattern, accounting for 6.67% of patients. All of the above descriptions are shown in Fig. 2.

The posterior tibial nerve branch ranged from 1.69 cm proximal to 3.78 cm distal to Line A. The average value of the bifurcation point was 1.56 cm distal to Line A. All of the bifurcation points were located above or inside the tarsal tunnel. The posterior tibial nerve was not found to branch distally to the lower border of the tarsal tunnel.

### Posterior tibial artery

**Figure 3:**
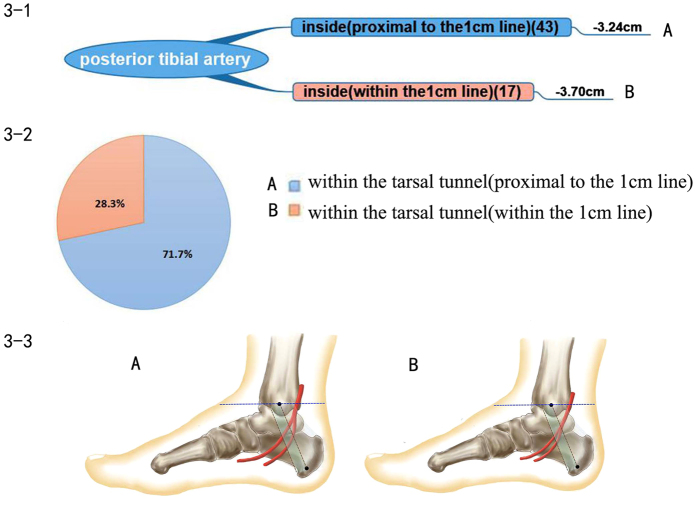
Posterior tibial artery. 3–1 Representation of the branch pattern of the posterior tibial artery. 3–2 Pie chart of the proportion of posterior tibial artery branch patterns. 3–3 Diagram of each branch pattern. (**A**) Type I: bifurcation point of the posterior tibial artery located within the tarsal tunnel, and proximal to the 1 cm reference Line B. (**B**) Type II: bifurcation located in the tarsal tunnel and in the area of the 1 cm reference Line B.

The posterior tibial artery was divided into two major branches, the medial and lateral plantar arteries with close accompanying veins on both sides. The bifurcation points of the posterior tibial artery were all located within the tarsal tunnel, and there were two types of branch patterns. Type I: bifurcation points of the posterior tibial artery were located within the tarsal tunnel proximal to the 1 cm reference Line B. There were 43 Type I cases in the 60 cases (71.67%). Type II: the bifurcation points were located within the tarsal tunnel and in the area of the 1 cm reference Line B, which is the end part of the tarsal tunnel. There were 17 Type II cases, 28.33% in total, as shown in Fig. 3.

The bifurcation point of the posterior tibial artery ranged from 1.83 cm distal to 4.72 cm distal to Line A. The average value was 3.37 cm distal to Line A.

### Position relationship between nerves and blood vessels

**Figure 4:**
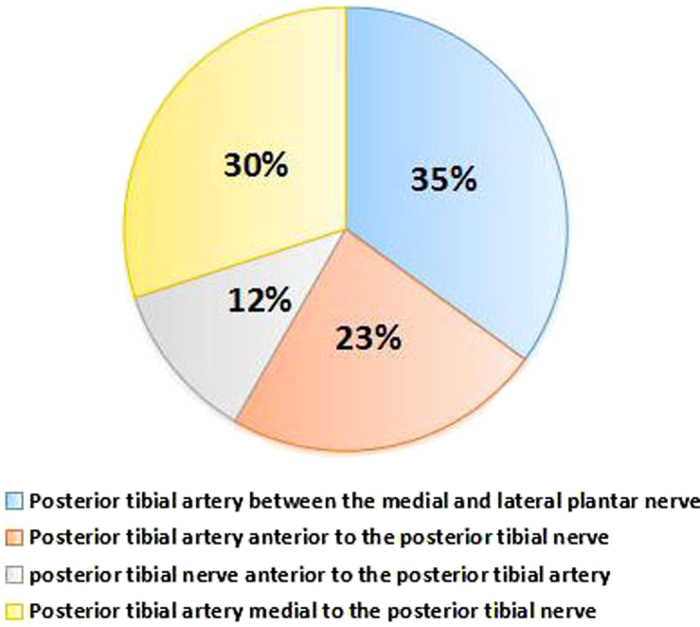
Relationship between the posterior tibial nerve and the posterior tibial artery.

The position relationship between the posterior tibial nerve and the posterior tibial artery was divided into four types^15^. For Type I, the posterior tibial artery was located between the medial and lateral plantar nerves, which occurred in cases of a high bifurcation of the posterior tibial nerve. There were 21 Type I cases (35%). For Type II, the posterior tibial artery was located anterior to the posterior tibial nerve. There were 14 Type II cases (23%). For Type III, the posterior tibial nerve was situated anterior to the posterior tibial artery. There were 7 Type III cases (12%). For Type IV, the posterior tibial artery was located medial to the posterior tibial nerve. There were 18 Type IV cases (30%), as shown in Fig. 4.

### Medial calcaneal nerve

**Figure 5:**
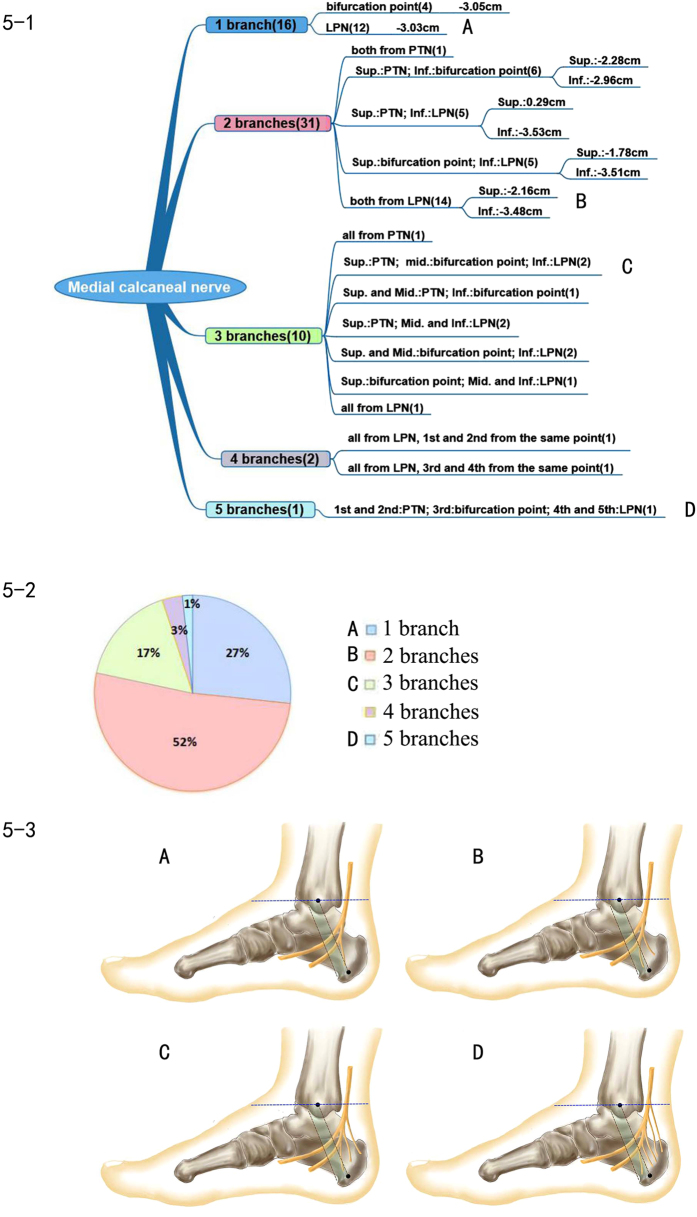
Medial calcaneal nerve. 5–1 Representation of the branch pattern of the medial calcaneal nerve. 5–2 Pie chart of the proportion of medial calcaneal nerve branch patterns. 5–3 Diagram of each branch pattern. (**A**) One medial calcaneal nerve branch. (**B**) Two medial calcaneal nerve branches. (**C**) Three medial calcaneal nerve branches. (**D**) Five medial calcaneal nerve branches.

The number and origination points of the medial calcaneal nerve were highly variable. The number of points ranged from one to five. In all 60 cases, there were 16 cases that had only one medial calcaneal nerve, 31 cases had two medial calcaneal nerves, 10 cases had three, 2 cases had four, and 1 case had five, with an average number of 2.02. In terms of the origination point, the medial calcaneal nerve could begin from the posterior tibial nerve, the lateral plantar nerve or the bifurcation point of the posterior tibial nerve. A medial calcaneal nerve that originated from the medial plantar nerve was not found. Figure 5 shows several major branch patterns.

The origin of the medial calcaneal nerve was highly variable, and ranged from 5 cm distal to 5 cm proximal to Line A. There was a case with five branches; the uppermost one originated from the posterior tibial nerve, 10.72 cm proximal to Line A, went into the tarsal tunnel deep to the flexor retinaculum and then innervated the heel.

### Medial calcaneal artery

**Figure 6:**
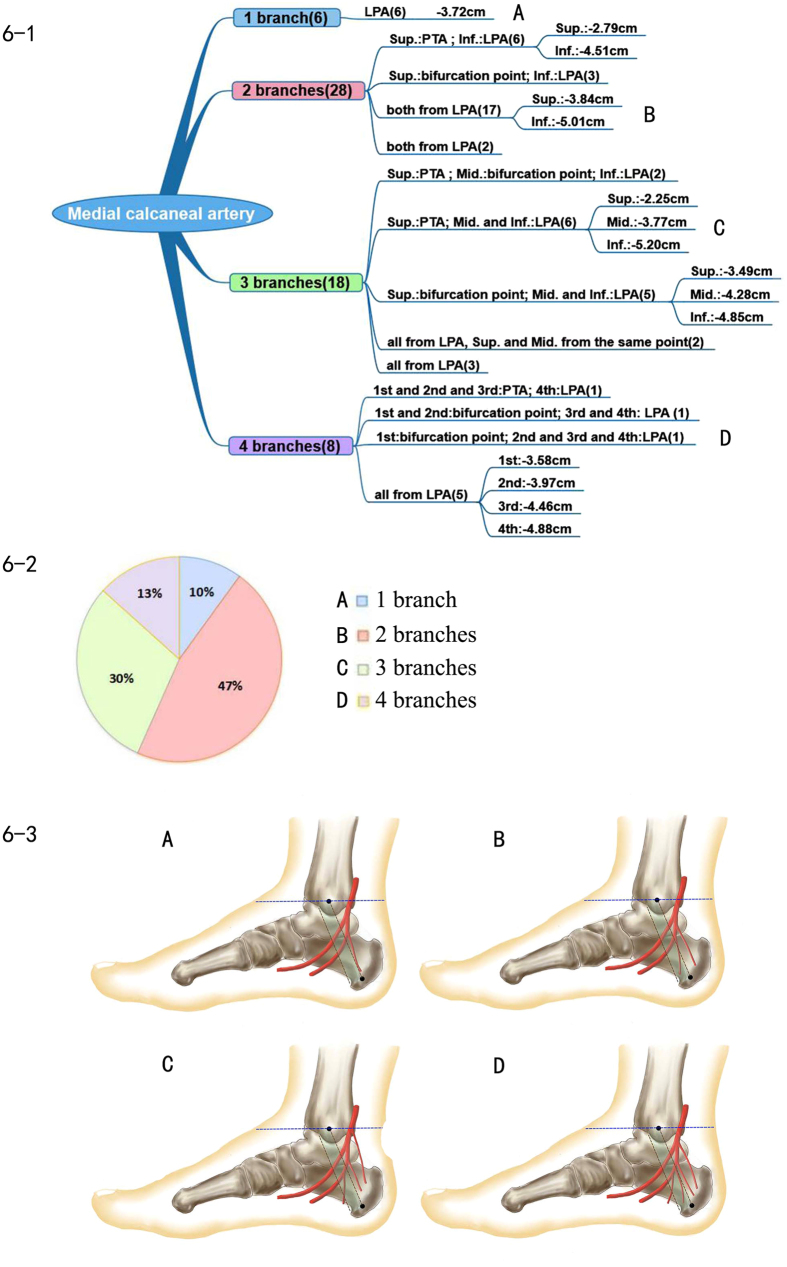
Medial calcaneal artery. 6–1 Representation of the branch pattern of the medial calcaneal artery. 6–2 Pie chart of the proportion of medial calcaneal artery branch patterns. 6–3 Diagram of each branch pattern. (**A**) One medial calcaneal artery branch. (**B**) Two medial calcaneal artery branches. (**C**) Three medial calcaneal artery branches. (**D**) Four medial calcaneal artery branches.

As seen in the medial calcaneal nerve, the number and the origin of the medial calcaneal artery were highly variable. The number of medial calcaneal arteries ranged from one to four. In 60 cases, one medial calcaneal artery was found in 6 cases, two branches were found in 28 cases, three in 18 cases, four in 8 cases, and the average number was 2.47. The medial calcaneal artery could begin from the posterior tibial artery, the lateral plantar artery or the bifurcation point of the posterior tibial artery. Figure 6 shows the main four branch pattern types.

The origin of the medial calcaneal artery was highly variable, but most of the arteries originated below Line A. In our studies, there was only 1 case with four branches; the uppermost branch originated 0.11 cm proximal to Line A from the posterior tibial artery. The remaining 59 cases were located in the tarsal tunnel below Line A. The lowest branch in the 59 cases originated from the lateral plantar artery 6.8 cm distal to Line A.

## Discussion

### Position and branch pattern of the bifurcation points of the posterior tibial nerve

There was a certain incidence rate of related diseases in the contents of the tarsal tunnel. Fine dissection of the nerves and blood vessels in this region is of great significance for clinical diagnosis and treatment. The study by Dellon and Mackinnond^10^ was similar to the study by Havel^12^. In all observed bifurcation points, 95% (Dellon) and 93% (Havel) of the tibial nerves were within 2 cm of the malleolar-calcaneal axis (a line passes the center of the medial malleolus and the center of the calcaneus). The malleolar-calcaneal axis was equal to the area of the tarsal tunnel. Bilge’s study^11^ showed that 96% of the bifurcations of the posterior tibial nerve were located within the tarsal tunnel, similar to the results of Dellon and Mackinnond and Havel. In the study by Bilge, 4% of the posterior tibial nerves branched distal to the tarsal tunnel. In our study, only 77% of the posterior tibial nerves were bifurcated in the tarsal tunnel; 18% were located proximal to the tarsal tunnel, and 5% were situated on the upper boundary of the tarsal tunnel. Bifurcation points located distal to the tarsal tunnel were not found. Kim^18^ of South Korea reported that the bifurcation point of posterior tibial nerve could be located as high as 1 cm proximal to the horizontal level of the tip of the medial malleolus. In our study, nearly 1/4 of the posterior tibial nerves had a high division, which was in contrast to previous studies from Europe and the United States. This discrepancy might be attributed to racial and/or individual differences.

### Position and branching pattern of the bifurcation points of the posterior tibial artery

We have clarified the position of the bifurcation points of the posterior tibial nerve. Whether the posterior tibial artery, which accompanies the nerve in the tarsal tunnel, has bifurcation points related to the posterior tibial nerve was not clear. We found that the bifurcation points of the posterior tibial artery were all located within the tarsal tunnel. Almost all bifurcation points of the posterior tibial artery were lower than that of the posterior tibial nerve. The average level of the bifurcation of the posterior tibial artery was 1.81 cm higher than that of the posterior tibial nerve. Only one case of bifurcation of the posterior tibial artery was 0.29 cm higher than that of the posterior tibial nerve. This confirmed the research of Bilge^11^ that the bifurcation of the posterior tibial artery is distal to the bifurcation of the posterior tibial nerve. However, in the study by Bilge^11^, the posterior tibial artery branched distal to the tarsal tunnel in 46% of cases, but this branch pattern was not found in our study. The four types of position relationships between the posterior tibial nerve and the posterior tibial artery could also provide evidence for related clinical operations.

### Position of origination and branch pattern of the medial calcaneal nerve

In previous papers, the description of the medial calcaneal nerve was similar: the number and the origination points were highly variable. Our study also confirmed this finding. Among the samples in the current study, two branches were the most common, followed by a single branch and three branches. We also found that there were two nerves that originated at the same point. A total of 17 types of branch patterns of the medial calcaneal nerve were found in our research. Havel^12^ found 9 modes of branch patterns. Dellon^13^ found 21 types of branch patterns. In our study, the medial calcaneal nerve appeared in all cases, and ranged in number from one to five. The medial calcaneal nerve can be initiated from the bifurcation point of the posterior tibial nerve, the posterior tibial nerve and the lateral plantar nerve. The origination points were very broad in scope, from 5 cm distal to 5 cm proximal to Line A. There was even a branch that originated from 10 cm above the horizontal reference line. Kim^18^ showed that the origin ranged from 1 cm distal to 3 cm proximal to Line A (an average of 0.2 cm proximal to Line A). Louisia^14^ applied the horizontal line of the bifurcation point of the posterior tibial nerve. The medial calcaneal nerve originated from 4 cm below to 10 cm above this line (average distance was 1.58 cm above this reference line). Dellon^13^ found that the medial calcaneal nerve could also originate from the medial plantar nerve, which crosses the superficial layer of the blood vessels within the tarsal tunnel to innervate the calcaneus. Havel^12^ also recorded this branch pattern. However, this was not observed in our experiment, and not all of the studies found or recorded such cases.

### Position of origination and branch pattern of the medial calcaneal artery

Research on the medial calcaneal artery is limited. The similarity between the medial calcaneal artery and the medial calcaneal nerve was that both the number and the origination points were highly variable. We found a range of one to four branches. The two branch pattern type was most common, followed by three branches. A total of 14 types of branch patterns were found. Two branches that originated from the same point were also observed. Just as the level of the bifurcation point of the posterior tibial artery was lower than that of the posterior tibial nerve, most of the levels of origination of the medial calcaneal artery were also lower than those of the medial calcaneal nerve. Only one medial calcaneal artery in all cases was found to originate above Line A, unlike the high origination of the medial calcaneal nerve. We speculated that the distribution of the medial calcaneal artery was the same as that of the medial calcaneal nerve. If disease or damage such as tarsal tunnel syndrome, calcaneal nail and clinical operation caused injury, it might trigger a change in the blood supply of the related region.

### Clinical relations

The tarsal tunnel is a closed structure that lacks flexibility. The entrapment of nerves, arteries and their branches within the tarsal tunnel is the cause of tarsal tunnel syndrome. Therefore, an understanding of the anatomical relationship between vascular and nerve components inside this limited space has important significance for clinical treatment. Excessive tension of the flexor retinaculum, deformity of the bones surrounding the tarsal tunnel, bleeding and adhesion caused by trauma, neuroma, space occupying lesions, even the placement of the foot in inversion and eversion positions, may cause neurovascular bundle compression^19^,^20^,^21^,^22^.

Extra attention should be paid to the main branches of the nerve and blood vessels to avoid iatrogenic injury during the operation. Surgical scar tissue may also cause a recurrence of tarsal tunnel syndrome^23^. Although it is used to relax the plantar fiber septum^24^, loosening the tarsal tunnel is still a key procedure. However, surgery carries a risk of injury to the nerves, and not all of the patients were satisfied with the results^25^. Therefore, trying to maintain the integrity of the neurovascular bundle is one of the essential factors of a successful operation. Some researchers have indicated that treatment of space occupying lesions are most likely to yield expected results after surgery^25^,^26^,^27^. Therefore, it is suggested that the space occupying lesion be regarded as the index of surgery^4^,^21^,^25^.

In this study, a large sample size of 30 cadavers of different ages with 60 cases were used. A horizontal line crossed with the tip of the medial malleolus was selected as a reference mark. The advantage was that this bony structure is relatively stable, does not arbitrarily slide, and can easily be palpitated. We described the branch pattern of the posterior tibial nerve and the posterior tibial artery and their corresponding positions on the basis of the reference line. These positions can provide an anatomic basis to prevent iatrogenic injury when performing a clinical operation, such as the external nailing of the tarsal bones, posterior tibial nerve block and surgery for tarsal tunnel syndrome^12^,^18^.

Medial calcaneal nerve injury was usually associated with heel pain. When performing surgery to remove the calcaneal spur and partial plantar fasciotomy, the operative incision was the site of medial calcaneal nerve crossing. Therefore, this nerve could easily be injured, which could lead to sequelae of heel pain due to the superficial nerve position^13^. In this study, we have described 17 types of branch patterns of the medial calcaneal nerve and 14 types of branch patterns of the calcaneal artery to provide more accurate positioning, which is a valuable reference for the clinic. In particular, the use of precise and detailed anatomic knowledge is helpful to prevent the injury of the nerve and the sequelae when performing calcaneal implant screw, calcaneal nerve block and foot surgery^13^,^14^.

## Conclusions

Combined with clear bony landmarks, two imaginary reference lines were chosen to identify the morphologic characteristics of nerves, blood vessels and their branches within the tarsal tunnel. The branch pattern and the corresponding position of them, such as lower bifurcation of the posterior tibial nerve than that of the posterior nerve, variable mode of the number and the origin of the medial calcaneal nerves and arteries, hinted that it was necessary to be cautious during decompression operation. Maybe only more above conditions were considered, the less complications, the higher the probability of successful surgery

## Methods

After the approval of the Ethics Committee of Dalian Medical University (DMU), 60 cases of 30 cadavers (left and right sides), which were donated through the Body Donation Center of DMU, were dissected in the Department of Anatomy of DMU. All methods were performed in accordance with the relevant guidelines and regulations established by the Ethics Committee. The age and gender distribution are shown in Table 1. Each foot was placed in the anatomical position that foot and the leg with an angle of 90 degree to standardize the measurement and image acquisition (Fig. 1). First, skin and adipose tissue of the medial foot were removed. Second, the flexor retinaculum was removed from the medial malleolus to the medial calcaneal tubercle. Finally, the posterior tibial nerve, posterior tibial artery and their branches were fully exposed.

A horizontal line that passed the tip of the medial malleolus (this is a point that is easily touched during biopsy) was regarded as reference Line A^18^. The distance from the bifurcation point of the posterior tibial nerve and that of the posterior tibial artery to the horizontal line were measured. The number of medial calcaneal nerves and the distance from origination points to the previous reference line were recorded. The data of the medial calcaneal artery were recorded and measured in the same way. Data distal to the reference line were recorded as negative, and data proximal to the reference line were recorded as positive.

Reference Line B with a 1 cm width was established from the tip of the medial malleolus to the medial tubercle of the calcaneus^11^, which was the lower boundary of the tarsal tunnel.

The position relationship between the posterior tibial nerve bifurcation point and the reference Line B was recorded, and the bifurcation point of the posterior tibial artery was also recorded. The measured data were input into the computer to make statistical charts and other representations. All pictures were drawn by easy paint tool SAI software (Systeamax Co., Japan) based on dissected specimens.

## Additional Information

**How to cite this article**: Yang, Y. *et al*. Fine dissection of the tarsal tunnel in 60 cases. *Sci. Rep.* **7**, 46351; doi: 10.1038/srep46351 (2017).

**Publisher's note:** Springer Nature remains neutral with regard to jurisdictional claims in published maps and institutional affiliations.

## Figures and Tables

**Table 1:** Age and gender distribution of cadavers.

Gender	Age (years)						Total
	20–29	30–39	40–49	50–59	60–69	70–79	
Male Cases	4	12	6	4	10	2	38
Female Cases	8	8	4	0	2	0	22
