# Author Correction: Fine dissection of the tarsal tunnel in 60 cases

**DOI:** 10.1038/s41598-021-91375-0

**Published:** 2021-05-28

**Authors:** Y. Yang, M. L. Du, Y. S. Fu, W. Liu, Q. Xu, X. Chen, Y. J. Hao, Z. Liu, M. J. Gao

**Affiliations:** 1 Medical Student in Clinical Medicine, Dalian Medical University, Dalian, 116044 People’s Republic of China; 2 Department of Anatomy, Dalian Medical University, Dalian, 116044 People’s Republic of China

Correction to: *Scientific Reports* 10.1038/srep46351, published online 11 April 2017

This Article contains an error in the legend of Figure 1, where the descriptions "posterior tibial artery" and "vein accompanying the artery" are swapped.

As such, the legend of Figure 1:

"MM: medial malleolus. MTC: medial tubercle of the calcaneal. The blue horizontal line is Line A, which crosses the tip of the medial malleolus. The green oblique line (band) is Line B, with a 1 cm width, which spans from the tip of the medial malleolus to the medial tubercle of the calcaneus. This axis also represents the inferior edge of the flexor retinaculum and consequently the tarsal tunnel. ① Posterior tibial tendon, ② flexor digitorum longus tendon, ③ posterior tibial artery, ④ vein accompanying the artery, ⑤ posterior tibial nerve, ⑥ flexor hallucis tendon."

should read:

"MM: medial malleolus. MTC: medial tubercle of the calcaneal. The blue horizontal line is Line A, which crosses the tip of the medial malleolus. The green oblique line (band) is Line B, with a 1 cm width, which spans from the tip of the medial malleolus to the medial tubercle of the calcaneus. This axis also represents the inferior edge of the flexor retinaculum and consequently the tarsal tunnel. ① Posterior tibial tendon, ② flexor digitorum longus tendon, ③ vein accompanying the artery, ④ posterior tibial artery, ⑤ posterior tibial nerve, ⑥ flexor hallucis tendon."

